# Augmented Reality Education Experience (AREduX): An Augmented Reality Experience and Experiential Education Medium to Teach Empathy to Healthcare Providers and Caregivers of Persons Living With Dementia

**DOI:** 10.7759/cureus.48384

**Published:** 2023-11-06

**Authors:** Gabrielle Hollaender, Eva Peisachovich, Bill Kapralos, Claire Culver, Celina Da Silva, Adam Dubrowski

**Affiliations:** 1 Information Technology/Health Education Technology Research Unit, Ontario Tech University, Oshawa, CAN; 2 Medical Education and Simulation, York University, Toronto, CAN; 3 Health Sciences, Ontario Tech University, Oshawa, CAN

**Keywords:** dementia, healthcare simulation, simulation, augmented reality, empathy

## Abstract

Previous research indicates that greater empathy by healthcare providers (HCPs) and informal caregivers leads to better care and improved patient satisfaction and outcomes for persons living with dementia (PLWD). Since few programs exist to train HCPs to develop empathy, we created the augmented reality education experience (AREduX), a proof-of-concept prototype that employs augmented reality (AR) to simulate the physical and cognitive symptoms that PLWD experience. This unique experience simulates the effects of dementia for training purposes with the goal of promoting more empathetic responses from HCPs and informal caregivers when attending to a PLWD. This technical report provides an overview of the five phases of the research program, conceptualization, development and design, usability testing and prototype updating, testing of refined prototype including measuring participants' empathy pre/post interaction with the AREduX, and analysis and dissemination of results, but focuses on Phase 2, development and design. We believe that the AREduX will substantially contribute to the scientific literature on the development of empathy, address the knowledge gap that exists regarding evidence-based understanding of empathy as a construct, and contribute to further recommendations aligned with implementing AR as an experiential education method to enhance empathy among HCPs and caregivers of PLWD.

## Introduction

While the evolution of healthcare has contributed to increasing the population's life expectancy, the number of individuals living with noncommunicable diseases such as dementia is on the rise. In 2015, the World Health Organization reported 47.5 million people living with dementia (PLWD) and postulated that this will increase to 75.6 million by 2030 [1]. Challenges that PLWD encounter include memory loss, depression, anxiety, isolation, inability to live independently, and loss of a sense of self [2]. Empathy, the ability to understand other people's feelings and perceptions, is a necessary component to caring relationships; it is, therefore, essential that it be embedded in the praxis of healthcare providers (HCPs). This is particularly true for caregivers of PLWD, whose success is linked with their ability to develop a trusting connection with their patient [3]. The two components of empathy, cognitive and emotional, are described as the ability to understand another's perspective and feeling another person's emotions, respectively [4]. Applying this theory to praxis can improve the professional relationship between caregivers and PLWD.

Simulation is defined as a mimicked representation of a natural phenomenon (a scenario) in a controlled environment. This technique can replicate substantial aspects of real-world experiences in a fully interactive, and often immersive, fashion. This training tool has increased in popularity [5] and is a well-established pedagogical practice in medical education [6]. Simulation types range from de-contextualized bench models and virtual reality (VR)- and augmented reality (AR)-based environments to high-fidelity recreations of operating rooms [7]. These virtual learning environments allow trainees to learn in an interactive, engaging, and ethically safe manner while allowing educators to offer simulated experiences to a more significant number of learners in a shorter period of time. The pedagogical use of digital technologies that include VR and AR has grown due to their advantages over traditional educational methods, as digital simulations provide these experiences in a cost-effective manner.

Research suggests that simulation is an appropriate methodology for developing empathy and/or empathetic behaviors in pre-service health professional students [8]. Immersive technology experiences have been shown to increase the user's physiological and emotional response and to captivate the user's attention [9]. These findings are not universal and are dependent on the type and educational features of the simulations and the definition of empathy and associated measures, collectively. Nonetheless, studies indicate that immersive digital technologies comprise an approach to simulation that seems most beneficial and is generally more effective in developing empathy, as it allows learners to literally stand in another person's shoes or act in the role of a patient. In the context of training and curricula development for HCPs, this may have significant implications for the educational design of simulations.

Further research is needed to confirm this result and to also investigate other types of simulation approaches, such as AR, and how they might promote or inhibit the development of empathy. The types of simulation approaches that have been used thus far include role-play, online training, virtual computerized interactive environments, and avatars. In order to learn which of these solutions would be most applicable to empathy-based training for HCPs, it was necessary to first gather a broad knowledge base from a variety of experts. With the goal of improving outcomes for PLWD, an interdisciplinary team of researchers with expertise in medicine/health sciences, medical education, computer science, and digital media gathered to examine the application of virtual learning environments for empathy-based training for HCPs and informal caregivers.

## Technical report

This research program involves the development of a proof-of-concept prototype in the form of a digital resource that uses AR to simulate the physical, cognitive, and sensory challenges that approximate those of PLWD. Team members involved in creating both prototype and scenario involve software and program developers, nurses, neurologists, and computer media and design experts. Using the latest AR and game development technology, we created a unique experience that provides simulating effects of dementia for training purposes with the goal of making HCPs and informal caregivers more empathetic to PLWD. The augmented reality education experience (AREduX) allows end users to complete tasks in their personal environment while wearing an "aging suit" (a suit that restricts the movement of knees, elbows, back, and neck, reduces sensitivity to touch, simulates blurry vision, and provides varying degrees of difficulty to represent the different stages of dementia). The AREduX will thus allow caregivers to build an understanding of the mobility and sensory issues that come with dementia, provide caregivers the appropriate training and confidence to care more effectively for PLWD, and allow researchers to explore how the medium impacts empathy levels among HCPs and caregivers of PLWD. Moreover, this educational component simulates a range of issues experienced by PLWD, from confusion and memory loss to physical signs (presbyopia, presbycusis, mobility issues, and psychomotor slowing).

Research methodology

Our interdisciplinary team of researchers with expertise in medicine/health sciences, computer science, digital media, and practicing medical professionals was formed to examine the application of immersive technologies to facilitate empathy-based training for HCPs and informal caregivers. Our team has extensive experience in simulation-based methodology research, evaluation research, mixed methods research, and research in application of experiential approaches to pedagogy which allowed us to develop, implement, and evaluate the AREduX as a means for developing empathy. This research is expected to contribute to enhancing theories and approaches in this area of study, allow for replication studies to further advance the integration of knowledge in this field, and contribute to our ultimate goal of improving outcomes for PLWD.

The AREduX research program involves five phases and is guided by the following research questions: (1) Are end users comfortable using the AREduX as an experiential education tool? (2) Does the AREduX enhance the empathy levels of HCPs working with PLWD? (3) Does the AREduX enhance empathy among caregivers of PLWD? (4) Will AREduX impact HCPs and caregivers' confidence levels when caring for PLWD?

In Phase 1 of the research program, a focus group involving a total of 12 participants including stakeholders, PLWD, HCPs, caregivers of PLWD, educational developers, and experts in computer science convened to support the preliminary development of the AREduX and to conduct a needs and task analysis [10]. A range of stakeholders was included among the participants ensuring various perspectives, but they broadly represented two subgroups. The first subgroup (n=7) comprised participants with experience living with dementia or caring for PLWD, while the second subgroup comprised educational developers and experts in computer science. All participants met the following inclusion criteria: able to understand, read, and speak basic English, willing to participate in a focus group, and able to access the internet. 

The focus group allowed us to gain insight into the elements to be employed in the AREduX. Key areas noted from the focus group included daily struggles related to caregiving; problems faced by PLWD when undertaking simple tasks such as grooming, feeding, and getting into bed; and symptoms associated with dementia such as memory loss and communication problems [10].

After conducting this focus group, our research group concluded that AR would be the most beneficial solution to address these outcomes [10]. This conclusion was aligned with creating a digital resource to expose users to an experience that gives them a sense of what it is like to live with dementia and suggested that using the latest game development technology could provide a unique experience that simulates the effects of dementia for training purposes with the goal of making users more empathetic. AR, as a technology, is able to provide and relate real-world stimuli, while VR is only able to provide simulations in a virtual world. As empathy and understanding are the goals for this simulation, we discussed whether a solely virtual environment could create possible dissonance with the user, depending on the level of fidelity. Fidelity refers to the degree of accuracy with which real-world experiences are reproduced in an interactive system [11]. To further assess the effectiveness of this solution, the AREduX was evaluated against other papers/projects that are similar in nature such as the use and impact of VR and AR simulation in dementia care and education training [8]. 

In Phase 2, data collected from stakeholder focus group during Phase 1 was used in conjunction with the design, play, and experience framework in the development of the AREduX prototype. The design, play, and experience framework is a formal approach in which the designers create a game to meet specific goals and the player plays the game [12]. The player's resultant experience can then be used to alter the design of the game to better achieve the targeted goals. Details of this design process will be further discussed in the next section.

Phase 3, which concluded in the summer of 2023, involved usability testing of the AREduX prototype with end users, including HCPs and caregivers of PLWD, to examine the functionality, clarity of content, ease of use, and user interface (results will be published in a separate article). Scales such as the System Usability Scale and the National Aeronautics and Space Administration (NASA) Task Load Index were used to analyze cognitive demand and whether participants would continue to use the system. This phase provided insights about the application of the technology and its potential within the user community and allowed us to examine and learn about its impacts within this context.

Phase 4, which will commence in the fall of 2023, will involve testing the refined AREduX version that was not captured by the usability study, with the goal of determining whether participants (a) wish to suggest changes or additions to enhance the educational experience design of the AREduX, (b) understand the purpose of the AREduX, and (c) use independent problem-solving while interacting with the AREduX. More specifically, Phase 4 will implement a pilot study in which 20 HCPs and 20 caregivers will receive AREduX training as an experiential education approach. Further, Phase 4 will measure participants' awareness and empathy levels via pre- and post-interaction questionnaires and a post-interaction focus group to determine whether empathy levels increase among HCPs and caregivers of PLWD who use the AREduX. The Sense of Competence in Dementia Care Staff (SCIDS) scale [13] will be used to determine whether the prototype impacts caregivers' confidence when they interact with PLWD.

Phase 5, in which data is analyzed and findings are disseminated, is ongoing and occurs after the completion of each phase. At the conclusion of Phase 4, a final analysis will be conducted. Success for this research program will be evaluated on two criteria: (a) the AREduX's efficacy as an education approach to enhance empathy and (b) the adoption of the AREduX into the training of HCPs and caregivers of PLWD, as well as into other departments.

Phase 2 development procedure

**Figure 1:**
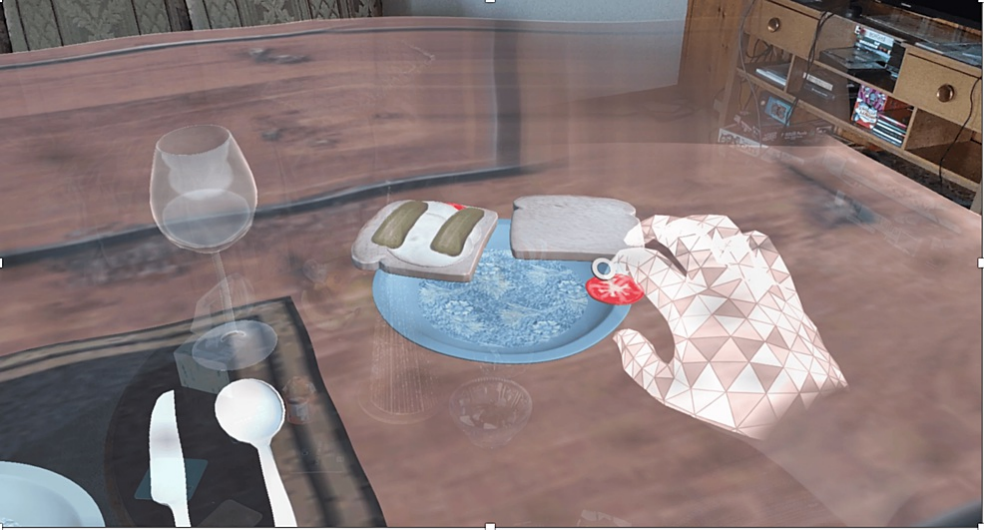
A screen capture of the scenario. The fluctuation of vision objects in the environment can be seen, along with a blurred area at the far corner of the table that is meant to simulate macular degeneration

Unity (Unity Technologies, San Francisco, California, United States), a game engine often used in academic contexts, was used to develop the AREduX simulation. Based on the outcomes of the focus group with stakeholders in Phase 1, a scenario was developed that involves the user setting a table for dinner (i.e., placing a plate, knife, and fork on the table), making a sandwich, and eating it as illustrated in Figure 1. 

The game logic was developed through repeated iteration cycles; after each iteration, play test sessions were conducted, feedback on the design and quality of the game logic was given by testers, and the feedback was used to further refine the simulation. In order to simulate some of the common visual impairments that can accompany the age where dementia presents, such as blurry vision, we leveraged the OpenVisSim library, a free resource that simulates a variety of vision issues (see https://github.com/petejonze/OpenVisSim). To ensure sustainability, the AREduX was designed so that it can be retained, reused, revised, adapted, modified, remixed, and redistributed.

To enhance the simulation, we added visual effects to simulate various visual impairments, such as macular degeneration, that are common in the age range that dementia presents in. Various scripts from the OpenVisSim library were implemented. In the case of macular degeneration, for example, we edited the script in question so that the displayed world blurred the farther away it was from the center of the effect. During testing, we found that having the effect cover a less easily defined space made for an equally frustrating experience, but caused less of an issue with breaking user immersion.

**Figure 2:**
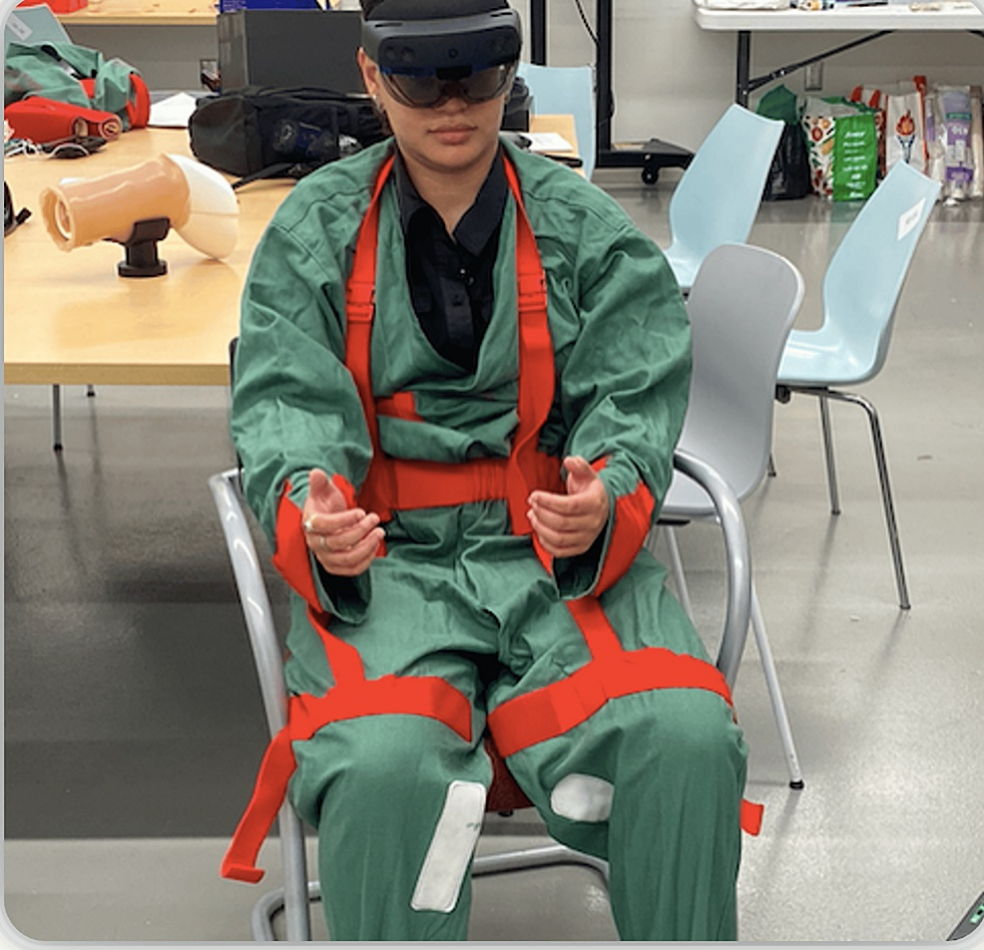
End user wearing aging suit while navigating through the AREduX prototype AREduX: augmented reality education experience

The game features were developed using Autodesk Maya 3D modelling software (Alias Systems Corporation, Toronto, Ontario, Canada). Each feature was modelled in a low-poly style to ensure lightweight compatibility. The Unity game engine was used to ensure that each feature has applied gravity physics to simulate realism. Dialogue was recorded and integrated so that each narrative segment is triggered at a specified point during the simulation. To further impact the comprehension of the user, as well as to artificially create frustration for the user, audio cuts out at certain portions. It is up to the user to determine their instructions based on the limited information that they are provided. This is intended to provide a possible empathy point for personal support workers who work with patients with aphasia. Aphasia impairs the expression and understanding of language and the ability to read and write, as there is damage to the brain that processes these functions [14]. While we were not able to mimic this phenomena in technology, we found this method of audio editing to be effective in providing a general sense of confusion even for those who do not have this disorder. Finally, to help build an understanding of the mobility and sensory issues that come with dementia, testers interacted with the AREduX while wearing an aging suit as illustrated in Figure 2.

## Discussion

Current support for PLWD relies on long-term care and local service centers to provide education and support. Caregivers and PLWD deserve to receive care from their HCPs that is supportive of their needs. Hence, HCPs who are equipped to provide quality care to PLWD are essential, and their training is of international concern. Further, there is increasing interest in developing approaches to aid patient care outcomes of PLWD and their caregivers. To achieve this goal, we must enhance the existing capacity of community-based care by creating resources for HCPs and caregivers that provide them with a deeper understanding of what it feels like to live with dementia.

AR-based programs continue to gain momentum across health sectors, as this innovative approach provides users an opportunity to have a visceral experience that can deepen their understanding and provide an embodied perspective of other groups within a relatively short time frame. The development and application of immersive digital technologies such as AR programs may represent one step in a process that enables HCPs to care empathetically for PLWD; however, further research is required.

Since there are little evidence about and few approaches for using AR in a healthcare setting, we believe that the AREduX, a digital resource that uses AR to simulate the physical and cognitive symptoms that PLWD experience, is vital to progress in the area of experiential education aligned with caring for PLWD.

In addition to its practical applications for HCPs caring for PLWD, the AREduX offers educational and scholarly advantages. Content can be reused for educational purposes for a wide array of learners including nurses, doctors, therapists, students, and administrators, as well as used at professional development training and workshops. As AR technology becomes more accessible and affordable, there is a unique opportunity to embed AREduX into the curriculum. Further, we believe that the data to be collected with the AREduX will (a) substantially contribute to the scientific literature on the development of AR protocols and related interventions, (b) address the knowledge gap that exists regarding an evidence-based understanding of empathy from the perspective of caregivers and HCPs of PLWD, (c) impact how empathy is conceptualized and taught within our curriculum and community, and (d) inform the development of research into and targeted approaches for implementing AR as an experiential education tool to enhance empathy among caregivers and HCPs of PLWD. Moreover, the AREduX can be integrated for various educational purposes, including professional development of HCPs and orientation and preparation prior to practicum experiences, and as part of academic curricula.

## Conclusions

The adoption of AREduX for training formal and informal caregivers has the potential to improve holistic care of PLWD by supporting the development of empathy in caregivers. This research program has significant expected outcomes for both academic and non-academic audiences. Our measure will contribute to the scientific literature on the development of empathy and may impact how we conceptualize and teach empathy within our curriculum and community.

Essentially, we aim to develop a greater understanding of whether the AREduX can be used as an effective education approach to impact the desired empathy outcomes. We are also interested in learning how ongoing incorporation of the AREduX into the training of HCPs and caregivers of PLWD can be adopted as a training approach or a professional development tool. AREduX will be the first digital training tool that provides an AR experience aligned with how it feels to live with dementia, a key factor in assuring the economic, social, and intellectual well-being of PLWD and their caregivers in Canada.
